# The reverse warburg effect in osteosarcoma

**DOI:** 10.18632/oncotarget.2352

**Published:** 2014-08-15

**Authors:** Federica Sotgia, Ubaldo E. Martinez-Outschoorn, Michael P. Lisanti

**Affiliations:** The Manchester Centre for Cellular Metabolism, The University of Manchester, UK; The Breakthrough Breast Cancer Research Unit, The University of Manchester, UK; The Manchester Centre for Cellular Metabolism, The University of Manchester, UK; The Breakthrough Breast Cancer Research Unit, The University of Manchester, UK

Osteosarcoma is a rare primary malignant tumor of the bone. It is a childhood cancer and has a peak incidence in the second decade of life. Unfortunately, osteosarcoma has a poor prognosis because of its metastatic dissemination to the bone and to the lung. In recent years, the 5-year survival for patients has increased to 60-70%, due to the development of multi-agent chemotherapy, used in conjunction with surgical techniques [1]. However, most current strategies have a limited efficacy in the treatment of metastatic and recurrent osteosarcoma. Hence, it is urgent to develop new strategies and innovative therapeutics, to further improve survival in patients with osteosarcoma. Although the exact cause of osteosarcoma remains unknown, certain potential molecular targets were discovered in recent years that might contribute to the treatment of osteosarcoma [2].

**Figure 1:**
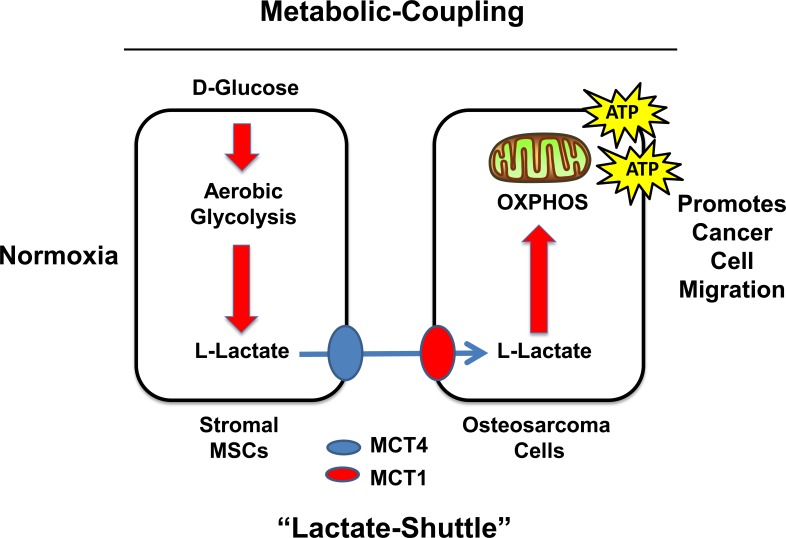
The role of MSCs and metabolic coupling in promoting the growth and migration of agressive osteosarcoma tumor cells.

In a recent issue of Oncotarget, Bonuccelli and colleagues [3] investigate the biological behavior of this aggressive cancer, by focusing on its metabolic aspect. The authors show that osteosarcoma cells drive the formation of two metabolic compartments within the tumor mass: i) tumor cells and ii) mesenchymal stem cells (MSCs), with mesenchyme being the origin of osteosarcoma cells. The authors demonstrate that, under aerobic conditions, that glycolysis (a.k.a., the Warburg effect) is induced in mesenchymal stem cells (MSCs) by adjacent osteosarcoma cells. Then, MSCs are able to “feed” osteosarcoma cells via their production and secretion of L-lactate, via the transporter MCT4. Importantly, tumor cells are able to import L-lactate, final product of glycolysis, via MCT1 expression, converting it to pyruvate and introducing it into the Krebs cycle (Figure 1).

It is now well-accepted by the scientific community that mitochondrial function plays a very critical role in tumorigenesis. Once again, these new findings support this assertion. In fact, Bonuccelli et al. show that in this scenario, oxidative phosphorylation (OXPHOS) and ATP-production are increased in osteosarcoma cells by co-culture with MSCs, and that the ultimate effect is to increase the aggressive behavior of tumor cells, in particular their cell migration. Intriguing, MSCs induce this phenotypic effect in osteosarcoma cells, due to oxidative stress.

Importantly, they also provide evidence for the existence of a shuttle for the extrusion of lactate in vivo. Indeed, in a xenograft model, MCT4 was over-expressed in the stromal compartment of human osteosarcoma tumors.

The existence of this metabolic-coupling between cancer cells and stromal components (cancer associated fibroblasts or MSCs) has been previously demonstrated in breast cancer, head and neck tumors, and prostate cancer [4-7]. Now, also in osteosarcoma, lactate seems to be one of the main players, and these current findings are consistent with the “Two-Compartment Tumor Metabolism Model” and “The Reverse Warburg Effect” [4-7].

Very likely, the reported metabolic reprogramming of MSCs causes not only the production of high-energy metabolites, such as lactate that fuels osteosarcoma cells and subsequent migration, but also increases the acidification of the tumor microenvironment, via the production and secretion of acidic metabolites, such as lactic acid. Importantly, it is well known that acidification promotes a metastatic phenotype, accelerating the degradation of the extracellular matrix.

Taken together, these findings suggest that lactate transporters (MCT family members, especially MCT1 and MCT4) are new attractive targets for more effective osteosarcoma therapy. In addition, novel therapeutic strategy should be used to simultanelously target both aerobic glycolysis in MSCs, as well as oxidative phosphorylation in tumor cells, to disrupt such metabolic coupling.
